# Psychophysiological Stress Markers and Behavioural Differences between Rural and City Primary School Students

**DOI:** 10.3390/ijerph17093157

**Published:** 2020-05-01

**Authors:** Daniel Mendoza-Castejón, Vicente Javier Clemente-Suárez

**Affiliations:** 1 Faculty of Sport Sciences, Universidad Europea de Madrid, 28670 Villaviciosa de Odón, Spain; daniel.mendoza@universidadeuropea.es; 2 Grupo de Investigación en Cultura, Educación y Sociedad, Universidad de la Costa, Barranquilla 080002, Colombia

**Keywords:** primary school, heart rate variability, stress, physical activity, nutrition, academic performance

## Abstract

Academic performance could be affected by multiple factors, including stress and learning environment location. The aim of this study was to analyze differences in psychophysiological stress markers, behavior and academic performance of rural and city students. A sample of 181 children (7.91 ± 2.29 years) from elementary schools were evaluated on their grades, subjective academic performance, heart rate variability, state anxiety, nutritional information and physical activity habits. Results presented significant higher values in parasympathetic modulation and physical education grades in rural students than in city students, who showed higher significant values in state anxiety, the ability to complete tasks, physical activity habits and several items relating to their food and drink habits. No significant differences were found in the average grades between the two groups. However, some correlations were found between school performance and stress, physical fitness and nutritional habits. Thus, school location may affect the stress and anxiety status, nutritional habits and physical activities of students, but there were no significant differences in academic performance. In addition, body mass index, quantity of food intake and stress markers may be related to the academic performance attained.

## 1. Introduction

Stress is an individual, natural and automatic reaction to a challenging or threatening stimulus. Its characteristic feeling of anxiety derived from the emotional response is associated with changes in the physiological and cognitive state [1,2]. It can evolve into a negative version (distress) and, when extended over time, it may be associated with serious pathologies. This fact is considered to be a big problem in modern western societies [3]. Focusing on the educational process, there are concrete situations to test students using daily training activities, evaluation tests or performance controls, which generate certain stress in students [4]. This fact motivated researchers to study the influence of stress on academic performance, applying different tests to be able to relate how this stressful state interferes in the achievement of educational objectives, even modifying the work of the internal brain structures. The stress generated in evaluative processes directly affect the synaptic efficacy and cortical plasticity, a fundamental mechanism in learning and memory function [5]. In higher educational stages, some stress sources like a fear of failure, work overload and a lack of self-confidence can limit the potential of achieving a strong academic performance [6,7]. Specifically at university, where students are subjected to anxiogenic formative situations such as clinical practices, anticipatory anxiety response and distress levels have been identified as a common problem [8,9,10]. Thus, it is important to highlight that in pre-adolescence, the mechanisms of regulation and control are not well developed; the prefrontal region of the cerebral cortex where executive function resides has not reached maturity yet, so concrete situations or stimuli that cause stress and anxiety can trigger a reduction in cognitive, memory and attention skills [11].

Elementary students are very sensitive to changes or situations that cause anxiety. Possibly, their external and internal demands regarding their performance are not as high as in later educational stages (secondary, preparatory or higher education), but they may also have a lack of self-control or suitable skills to manage this type of situation. The state of anxiety and stress levels seem to increase as the academic years progress, even showing psychosomatic responses in students [12]. The personal contribution to the classroom climate and individual academic performance may be affected by changes in the psychophysiological state [12], although the direct influence of stress on grades was not widely covered in any specific literature in depth, which was one of the motivations for carrying out this study. Previous research into different educational stages have been focused on the relationship between stress and academic performance with other variables. Elements of an internal nature such as psychological and emotional control [13], self-demand in terms of expectations, biogenic diseases or unhealthy daily habits such as a lack of physical activity [14], lack of sleep, poor diet or excessive time in front of the screen [15] have been pointed out as being influential elements. Regarding external influences, schedules and routines that are too stifling, a lack of time to do homework/study, parental pressure to reach a certain grade and an excessive amount of school activities could make teachers perceive that students are more nervous and distant with a higher stress perception, facts which contribute to worsening the student–teacher relationship [16,17]. Furthermore, the state of anxiety in students could also be transmitted by the teacher’s exhaustion and their work stress [18].

Among the external factors previously mentioned, we could add the location of the educational center and personal residence [19], which could present urban secondary students with higher stress levels [20]. In fact, it seems that stressors in the urban area could decrease the neural process by modifying the function and structure of the medial prefrontal cortex (mPFC) grey matter volume, a basic structure to achieve academic performance [21]. Schools located in an urban area presented some stressful physical characteristics such as noise, pollution, artificial light, small and unnatural spaces or unhealthy transport routes [22,23,24,25,26]. In addition, if there is a constant environment of competitiveness or rapidly covered needs, a certain feeling of non-frustration tolerance and dissatisfaction materializes from an early age, which increases the feeling of anguish, anxiety, dependency, anger or aggressive behavior in urban children [27,28]. In a rural area, students may suffer from a lack of means of teaching [29], but the environment could make it appear active and there is likely to be fewer pressuring methodologies, reducing anxiety and increasing the well-being of students in the classroom [30].

The aim of this study was to analyze the differences in the psychophysiological stress markers, nutritional and physical activity habits, and the academic performance of rural and urban students. The initial hypothesis was that students in rural schools would present fewer stress markers and healthier behaviors in terms of healthy nutrition and the amount of daily physical activity. The rural group would present a higher academic performance according to the previous arguments. 

## 2. Materials and Methods

### 2.1. Participants

One hundred and eighty one children (7.91 ± 2.29 years) from pre-school and primary school were analyzed (ranging from 3 to 12 years). This sample was composed by students from two different public schools. The first one was a rural school placed in a village with less than 1000 residents (Rural School Group, RSG *n* = 75), and the second one was a school in a city with over 100,000 residents (City School Group, CSG; *n* = 106). The sample size obtained was in accordance with a confidence level of 99% and a margin of error between 8%–9% (total rural school students: 115; urban shool:172). Dividing the sample by center and sex, we observed that the RSG was made up of 54.7% (*n* = 41) male students and 45.3% female (*n* = 34). The CSG was 48.1% (*n* = 51) male and 51.9% (*n* = 55) female. Related to the division by school year, there were as follows: pre-school education (3–5 years), RSG: 11, CSG: 16; first primary stage (1st and 2nd, 6–7 years), RSG: 27, CSG: 31; second stage (3rd and 4th, 8–9 years), RSG: 19, CSG: 32; third stage (5th and 6th, 10–12 years), RSG: 18, CSG: 27. All the data were collected anonymously. All the procedure was approved by the University Ethical Committee. Prior to participation, all participants, parental or guardian and their professors were informed about the experimental procedures, indicating the right to withdraw from the study at any time and providing a written informed consent following the Helsinki Declaration (as revised in Brazil, 2013).

### 2.2. Procedure

We analyzed psychophysiological stress markers, behavior and academic performance in two student groups from two different schools located in different Spanish regions. Consulting data from the National Statistics Institute of Spain (INE), the first location is Pueblonuevo de Miramontes (Rural School Group), a small town in Caceres, Extremadura. In 2018 it had 814 inhabitants (51.2% men, 48.8% women), with an average age of 43 years (17% under 18 years and 18% over 65 years). Pueblonuevo has essential public services. The average income per family was around 19,000 Euros. Its main economic activity is agriculture, which is located in the primary economic sector. There is only one school in the city. By contrast, Fuenlabrada (City School Group) has 51 schools, 39 of them are public institutions. Fuenlabrada is located in the Madrid region (the third most populous Spanish community) but very close to the capital, Madrid, which the most populous city in the country (about 3,270,000 inhabitants). Fuenlabrada had 193,700 inhabitants (49.3% men, 50.7% women) in 2018. Their average age was 38.9 years, with 20% of children under 18 years and 10% over 65 years. The town had an income close to 30,000 Euros. This city has public services like hospitals and higher education centers. Its fundamental activity is industry and services, belonging to the second and third economic sectors. In fact, it has one of the largest business centers in the European Union.

The data collection took place in the last week of the first term evaluations and before the report cards were given. Both schools followed the national curriculum and although there is a different territorial regulation, the contents and evaluative tests were verified as being similar in terms of workload and evaluation systems without differences.

The week previous to data collection, a visit was made by the researchers to introduce themselves to the children and to show the devices used for the data collection to familiarize them and to reduce possible anxiety at the time of measurement. At this point, they were helped by the teachers from both centers. Furthermore, the students who participated in the study had filled out a small booklet two weeks before to collect hear rate variability (HRV) data at the school. They were helped by their parents and teachers, especially at the earliest ages. This document contained their basic data and the questionnaires specified below. Likewise, the center provided the students’ grades with their express authorization. 

The autonomic modulation was evaluated by an analysis of heart rate variability (HRV). An R-R waves interval of the heartbeat, used as a measure of autonomic modulation, was recorded with a Polar V800 heart rate monitor (Polar, Kempele, Finland), consistent with previous research [31]. The R-R series was analyzed using the Kubios HRV software, version 2.0 (Biosignal Analysis and Medical Imaging Group, University of Kuopio, Finland), developed in accordance with the recommendations of the existing scientific literature [32]. The following physiological measurements were evaluated: the low-frequency band in normalized units (low-frequency LFn); the high-frequency band in normalized units (high-frequency, HFn); percentage of differences between normal adjacent R-R intervals greater than 50 ms (PNN50); the square root of the average of the sum of the differences squared between normal adjacent R-R intervals (RMSSD); heart rate; and the sensitivity of the short-term variability (SD1) and the long-term variability (SD2) of the non-linear spectra of the HRV. We analyzed the HRV for five minutes prior to the start of a regular school day, with the students sat in a quiet room, following previous procedures [33,34].

The Physical activity was analyzed with a validated self-report questionnaire in children and adolescents, PAQ-C and PAQ-A, respectively [35]. Body Mass Index (BMI) was calculated using the classic formula: weight(kg)/height(m)^2^.

Nutrition habits were measured by a questionnaire that was administered to the parents to fill in with the habits of their children. The survey consisted of 16 questions, the first five with dichotomous answers (“yes or no”) and the rest with questions ranging from 1 to “more than 5”:

1. How many meals do you eat a day?

2. How many pieces of fruit and vegetables do you eat daily?

3. How many dairy products do you eat daily?

4. How many times a day do you eat sweets, snacks or industrial pastries?

5. How many times a week do you eat meat?

6. How many times a week do you eat fish?

7. How many times a week do you eat legumes?

8. How many times a week do you eat “fast food”? 

9. How many soft drinks do you have per week?

10. How many times a week do you eat “fried foods”?

11. How many glasses of water do you drink a day?

Academic performance was analyzed by the academic mark of the different subjects and the average of the marks in all the subjects. In addition, teachers filled in a questionnaire to analyze their subjective perception of students’ academic performance, wherein they answered the following 5 questions in a rank between 1 (never) and 5 (always):

1. Student shows interest and curiosity in learning new things.

2. Student works to finish the tasks you begin.

3. Student stays calm and in control when faced with a challenge.

4. Student cares about doing well in school.

5. Student does all the work required. 

Anxiety was measured by the State-Trait Anxiety Inventory for Children and Adolescents (STAIC) [36]. It is composed of two scales, the first one to measure State Anxiety, listing 20 items, and the second one to measure Trait Anxiety with 20 items.

### 2.3. Statistical Analysis

The statistical analysis was carried out using the Statistical Package for the Social Sciences (SPSS), version 22.0 (SPSS Inc., Chicago, Ill., USA). Descriptive statistics (mean and standard deviation) were calculated for each variable. The normality was tested by the Kolmogorov–Smirnov test. As all the variables presented a parametric distribution, an independent t-test was carried out. The correlation analysis was performed by the Pearson’s test. The Effect Size was calculated by Cohen´s D. The significance level was 0.05. 

## 3. Results

We found significantly higher PNN50 in RSG than in CSG (Table 1). The RSG had significantly higher height and physical education grades than CSG. On the contrary, CSG presented a significantly higher state of anxiety in their ability to finish the work tasks they had started, in their physical activity habits (Table 2) and in ten of sixteen items related to their daily and weekly food and drink intake (Table 3) than the RSG.

The correlation analysis showed a negative significant correlation between BMI and grades average (*r* = −0.288, *p =* 0.010), social sciences grade (*r* = −0.210, *p* = 0.017), language grade (*r* = −0.195, *p* = 0.028), English grade (*r* = −0.220, *p* = 0.013), arts grade (*r* = −0.198, *p* = 0.025), visual arts grade (*r* = −0.190, *p* = 0.031), religion/social values grade (*r* = −0.257, *p* = 0.004) and in teachers’ subjective perception of the academic performance of students (*r* = −0.181, *p* = 0.042). Moreover, negative significant correlation was found between teachers’ subjective perception of the academic performance of students and RMSSD (*r* = −0.191, *p* = 0.027), PNN50 (*r* = −0.283, *p* = 0.001) and SD1 (*r* = −0.191, *p* = 0.027).

Positive significant correlations were found in “How many meals do you eat a day?” and grades average (*r* = 0.265, *p* = 0.001), mathematics grade (*r* = 0.246, *p* = 0.002), English grade (*r* = 0.270, *p* = 0.001), arts grade (*r* = 0.217, *p* = 0.008), music grade (*r* = 0.229, *p* = 0.005) and teachers’ subjective perception of the academic performance of students.

## 4. Discussion

The aim of this study was to analyze the differences in the psychophysiological stress markers, nutritional and physical activity habits and academic performance of rural and urban students. The initial hypothesis was partially confirmed since rural students presented lower stress values than city students, but there were no significant differences related to academic performance except for grades in physical education. By contrast, city students showed higher values in some of the nutritional parameters and the weekly amount of physical activity, which differed to the initial hypothesis.

### 4.1. Stress, Anxiety and Center Location

We found how CSG presented a significant lower PNN50 than RSG with a medium effect size, showing a higher sympathetic modulation as previous authors have reported [37]. These data obtained by CSG evidence a higher physiological stress response, and the higher state anxiety in the city students shows a higher psychological stress compared with rural students. School location and environment may be important factors in modulating the stress response in this young population [38,39,40].

Along this line, an important influential factor is the center conditions: facilities, classroom saturation, insufficient material resources due to a high ratio of student or disaffected/depersonalized teaching. These changes usually differed in cities from small towns. In the case of the two schools analyzed, they were small centers with similar means, but in the last few years, the ratio of students increased in the city school and the attitude of these teachers towards students became more severe and directive. Rural schools are characterized by a small number of students per class, a more familiar treatment by staff, and a general environment that is more integrative with the social environment and the community [41]. The specific location is also something to review. Having large spaces without buildings nearby and a more relaxed environment is not the same as being in the middle of strong stressors with pollution, noise, artificial light, heavy traffic or being faced with aggressive and competitive situations [42,43]. Climate and severe pollution in school areas could also affect children of school age [44]. In this case, the urban school was in the center of large blocks of flats, surrounded by large ring roads with major parking and public transport problems. However, the rural school was close to low buildings and a large agricultural/natural area, where the level of traffic was very low, allowing families to walk and children to play safely. Environmental conditions directly affect lifestyle, organic systems, and the stress response of adult citizens [45,46], generating situations that cause irritability, anxiety and insomnia, leading to physical illness or emotional problems, as well as a lack of concentration and poor performance. All these symptoms and consequences can be easily transferred by families and teachers to the child and adolescent school population [47]. In this sense, the atmosphere breathed in the rural school environments visited was much more relaxed compared to the city school.

### 4.2. Academic Performance, Body Composition and Physical Activity

There were no significant differences in academic performance between the two groups of students, showing how the location of the center presented no effect (except in physical education where the RSG obtained better grades). The higher physical education mark of the RSG could be related to the fact that living in a rural location seems to be associated with unregulated physical activity habits, obtaining spontaneous multilateral development that could help to adapt to the demands in basic skills and abilities demanded by physical education in preschool and primary education [48,49]. However, the RSG presented significantly less weekly physical activity than the CSG. This contradiction could be interpreted as being due to fewer hours of sports activity in a classic format (guided classes and training) compared to spontaneous physical activity that could occur in rural areas, such as the usual forms of transportation to school, games in free time, or help with family jobs at home or in the fields. Regulated extracurricular physical activities with a closed structure (modality and practice time) and a classic format are more regular and demanding, but are also more specialized and are makers of daily habits. Depending on the area, there are different things on offer, conditioning the practice [50]. This type of practice is better suited to the urban lifestyle, where the need to adhere to a strict plan and schedule is essential for family organization and is much more pronounced due to the overload of daily activities [51,52]. For that reason, this habit might also explain the higher values achieved by the CSG in response to quickly completing the tasks committed.

After the evaluation of body composition and academic performance, the RSG presented a higher height (but similar BMI) compared to the CSG group. This may be related to a greater physical development in the anthropometric variables that are being observed in the rural environments of developed countries [53,54,55].

### 4.3. Nutritional Habits

Regarding nutritional habits, we found in the CSG a significantly higher intake of food and beverages in most of the items, both in terms of quantity and variety of products. However, the RSG had a more relaxed and calm way of consuming food, coinciding with some arguments that have been explained in the previous paragraph. The results obtained by CSG are consistent with healthy eating and hydration (intake of fruit and vegetables, fish, pulses and water consumption), but they also reflect a high consumption of sugar and saturated fat (fast food, dairy, soft drinks or meat). No differences were found between the two groups at this point, as currently observed in the behaviors of children and youth globally [56]. These nutritional trends found in city and rural students have also being observed in countries with a traditional culture of healthy foods, such as Mediterranean countries [57]. This fact caused real alarm in all countries and is aggravated when children pass into adolescence, with more autonomy to be able to access unhealthy foods, and when the amount of physical exercise is reduced, especially in women [58,59]. Possibly the great offer made by these products and the change in leisure-time habits and a more sedentary lifestyle (life spent in the mall, time in front of screens, use of social networks, etc.), require a change in nutritional strategy and an increased amount of physical activity [60].

### 4.4. Main Correlations

In the correlational analysis we found a negative correlation between BMI and the average grades and subjects not related with mathematics and science (language, socials, English, arts, visual arts and religion/social values). Higher BMI values have been negatively correlated with subjective perception and objective academic performance data according to other studies [61,62]. Similarly, we also found this negative correlation with some of the heart rate variability markers. According to these results, a body composition classified as unhealthy would be negative in an educational environment, directly affecting both the level of performance, as well as the adaptability of the student’s parasympathetic system to adequately modulate the stressful situations that occur in this environment [63]. It seems necessary to find ways to implement school and extracurricular programs to improve the physical condition supported by a good nutritional strategy because non-healthy habits are enveloping children and youths in all demographic areas [64]. A concentrated program applicated after identifying the problem would place the students in a better situation to face daily educational challenges [65].

A significant negative correlation has been shown between the subjective perception of academic performance of teachers and sympathetic modulation. A better performance in tasks and daily formative actions was related to higher values of stress; there is a possibility that reaching the best-expected standards will place greater stress on students [66,67,68]. The values in the variables that were presented within these groups of students (RMSSD, PNN50 and SD1) were above the average by the age established in previous studies [69], showing a normalized parasympathetic modulation [70] according to the evolution of heart rate variability with age [71]. We also found a significant positive correlation between the number of meals made per day and the average grade, teachers’ subjective perception of students’ academic performance and some specific subjects (mathematics, English, arts and music). This fact is in accordance with previous studies that show how a greater number of meals per day of an appropriate quality that resolve children’s demands might improve physical and physiological growth and academic performance [72,73].

## 5. Limitations 

The main limitation of the study was the small sample size and it was difficult to get centers to participate fully in all the research requirements. A salivary analysis of amylase or cortisol samples could have allowed an analysis of the hormonal stress response, but the lack of material and economic resources prevented its collection. Future research could be implemented to complete the physiological stress profile

## 6. Conclusions and Practical Applications

School location is a variable that can affect anxiety levels and ability to modulate the internal stress of students. City students had higher anxiety scores and higher records on variables related to sympathetic system activation than rural students. However, no differences in academic performance were observed (excepting the higher mark of rural students in physical education). Regarding eating habits, there was a significant difference in relation to the amount of daily intake of city students but not in terms of food/drink quality that can be explained by having greater opportunities and a greater offer and variability of products to consume. The results show that a worse body composition (associated with an unhealthy physical condition) correlates with lower grades. Likewise, academic performance does not mean better control or less stress. The amount of daily food intake was positively correlated with performance and grades, possibly due to greater energy needs during growth and intense study. Proposals to implement programs to improve healthy nutritional habits, practice physical activity and control stress in two basic dimensions of action (curricular and extracurricular) could improve basic conditions in order to better face daily school demands. These interventions should be implemented by political, educational and family institutions to promote positive behaviors and decrease anxiety in the school environment. 

## Figures and Tables

**Table 1:** Heart rate variability results of rural and city school students.

Variables	RSG	CSG	T	*p*-Value	95% Confidence		Cohen’s D
	*n* = 75	*n* = 106			Lower	Upper	
**HRmean**	105.37 ± 61.91	98.74 ± 13.15	0.976	0.331	−6.79	20.05	−0.11
**RMSSD**	67.00 ± 25.59	61.25 ± 36.02	1.091	0.277	−4.66	16.15	−0.22
**pNN50**	30.33 ± 15.52	23.27 ± 15.44	2.777	0.006	2.03	12.08	−0.45
**LF**	67.99 ± 16.78	64.12 ± 16.48	1.426	0.156	−1.50	9.33	−0.23
**HF**	30.87 ± 16.27	35.71 ± 16.38	−1.808	0.073	−10.21	0.45	0.30
**LF_HF**	4.23 ± 5.42	2.60 ± 2.40	1.413	0.160	−3.60	14.70	−0.15
**SD1**	47.43 ± 18.12	43.36 ± 25.50	1.092	0.277	−2.92	11.06	−0.22
**SD2**	121.91 ± 70.66	112.46 ± 57.34	0.910	0.365	−11.81	30.72	−0.13

RSG, rural school group; CSG, city school group; HRmean, heart rate mean; RMSSD, root-mean square differences of successive heartbeat intervals; pNN50, percentage of successive RR-interval pairs differing in more than 50 milliseconds in the entire recording divided by the total number of RR waves intervals; LF: low-frequency band; HF: high-frequency band; LF/HF Ratio; SD1, transverse axis; SD2, longitudinal axis.

**Table 2:** Body composition, academic performance and physical activity habits of rural and city school students.

Variable	RSG	CSG	T	*p*-Value	95% Confidence		Cohen’s D
	*n* = 75	*n* = 106			Lower	Upper	
Height	139.05 ± 13.53	132.50 ± 16.05	2.29	0.023	0.89	12.20	−0.48
Weight	35.94 ± 9.69	33.75 ± 12.47	1.061	0.290	−1.89	6.27	−0.23
BMI	18.62 ± 3.03	18.66 ± 3.89	−0.63	0.950	−1.39	1.30	0.01
Grades_Average	7.12 ± 1.31	7.00 ± 1.13	0.605	0.546	−0.27	0.51	−0.09
Sciences_Grade	6.96 ± 1.63	7.18 ± 1.47	−0.87	0.386	−0.72	0.28	0.13
Social sciences_Grade	6.73 ± 1.79	6.55 ± 1.64	0.622	0.535	−0.37	0.72	−0.10
Lenguage_Grade	7.33 ± 1.69	6.98 ± 1.64	1.256	0.210	−0.19	0.88	−0.21
Mathematic_Grade	7.15 ± 1.83	6.91 ± 1.66	0.87	0.386	−0.31	0.81	−0.13
English_Grade	6.09 ± 2.05	6.42 ± 1.70	−1.071	0.286	−0.93	0.27	0.16
Arts_Grade	7.15 ± 1.32	7.33 ± 1.18	−0.837	0.395	−0.57	0.22	0.14
Music_Grade	7.01 ± 1.48	7.08 ± 1.39	−0.310	0.757	−0.53	0.39	0.05
Visual arts_Grade	7.36 ± 1.39	7.04 ± 1.39	1.567	0.119	−0.08	0.72	−0.23
Physical education_Grade	7.71 ± 0.96	6.68 ± 0.91	6.331	0.000	0.70	1.34	−1.07
Religion/S-C Values_Grade	7.90 ± 1.18	7.82 ± 1.36	0.388	0.698	−0.33	0.50	−0.07
SPAP_Average	3.74 ± 1.21	3.86 ± 0.94	−0.7	0.485	−0.46	0.22	0.10
Interest and curiosity in learning new things	4.07 ± 1.70	3.81 ±1.06	1.195	0.234	−0.16	0.69	−0.15
Works to finish the tasks you begin	3.50 ± 1.95	3.98 ± 1.10	−2.004	0.047	−0.95	−0.00	0.25
Stays calm and controlled when faced with a challenge	3.57 ± 1.93	3.65 ± 1.20	−0.322	0.748	−0.56	0.40	0.04
Student cares about doing well in school	3.85 ± 1.82	3.84 ± 1.15	0.034	0.973	−0.45	0.47	−0.01
Student does all the work required	3.71 ± 1.88	4.02 ± 1.08	−1.348	0.180	−0.77	0.14	0.16
PAQ-A	1.06 ± 0.50	1.99 ± 0.35	−14.549	0.000	−1.05	−0.80	1.86

BMI, Body Mass Index; S-C, Social and Civic; SPAP, Subjective Perception of Academic Performance; PAQ-A: Physical Activity Questionnaire for Adolescents.

**Table 3:** Anxiety and nutritional information of rural and city school students.

Variables	RSG	CSG	T	*p*-Value	95% Confidence		Cohen’s D
	*n* = 75	*n* = 106			Lower	Upper	
State Anxiety	26.53 ± 4.90	29.80 ± 3.23	−5.399	0.000	−4.46	−2.07	0.67
Trait Anxiety	24.17 ± 5.65	25.56 ± 4.60	−1.814	0.071	−2.90	0.12	0.25
Do you think you have a healthy diet?	0.94 ± 0.23	0.85 ± 0.35	1.83	0.069	−0.00	0.17	−0.39
Do you grab snacks between hours?	0.59 ± 0.49	0.44 ± 0.49	1.942	0.054	−0.00	0.29	−0.31
Do you follow any kind of diet?	0.19 ± 0.39	0.16 ± 0.36	0.585	0.559	−0.08	0.15	−0.08
Do you read food labels to know their composition?	0.27 ± 0.45	0.27 ± 0.44	0.061	0.951	−0.13	0.13	0.00
Do you eat slowly and while sitting?	0.94 ± 0.22	0.84 ± 0.35	2.018	0.045	0.00	0.19	−0.45
How many meals do you eat a day?	3.67 ± 0.75	4.49 ± 0.81	−6.691	0.000	−1.06	−0.57	1.09
How many pieces of fruit and vegetables do you eat daily?	1.66 ± 1.39	2.46 ± 1.45	−3.541	0.001	−1.23	−0.35	0.58
How many dairy products do you eat daily?	1.81 ± 1.36	2.67 ± 1.23	−4.353	0.000	−1.25	−0.47	0.63
How many times a day do you eat sweets, snacks or industrial pastries?	1.31 ± 1.78	1.61 ± 0.94	−1.421	0.157	−0.70	0.11	0.17
How many times a week do you eat meat?	2.32 ± 1.51	3.31 ± 1.22	−4.774	0.000	−1.39	−0.57	0.66
How many times a week do you eat fish?	1.41 ± 1.56	2.50 ± 1.20	−5.16	0.000	−1.39	−0.57	0.70
How many times a week do you eat legumes?	1.57 ± 1.54	2.36 ± 1.11	−3.95	0.000	−1.18	−0.39	0.51
How many times a week do you eat "fast food”?	0.95 ± 1.50	1.57 ± 1.10	−3.115	0.002	−1.01	−0.22	0.41
How many soft drinks do you have per week?	1.35 ± 1.63	1.97 ± 1.36	−2.666	0.008	−1.07	−0.16	0.38
How many times a week do you eat “fried foods”?	1.74 ± 1.55	2.14 ± 1.23	−1.852	0.066	−0.81	0.02	0.26
How many glasses of water do you drink a day?	3.85 ± 1.43	4.67 ± 1.43	−3.73	0.000	−1.25	−0.38	0.57

## References

[1] Sánchez-Conde P., Beltrán-Velasco A.I., Clemente-Suárez V.J. (2019). Influence of psychological profile in autonomic response of nursing students in their first hospital clinical stays. Physiol. Behav..

[2] Maslach C., Schaufeli W.B., Leiter M.P. (2001). Job burnout. Annu. Rev. Psychol..

[3] Eurofound, EU-OSHA (2014). Psychosocial Risks in Europe—Prevalence and Strategies for Prevention.

[4] Beltrán-Velasco A.I., Bellido-Esteban A., Ruisoto-Palomera P., Mendoza K.H., Clemente-Suárez V.J. (2020). The effect of cultural differences in psychophysiological stress response in high education context: A pilot study. Appl. Psychophysiol. Biofeedback.

[5] Concerto C., Patel D., Infortuna C., Chusid E., Muscatello M.R., Bruno A., Zoccali R., Aguglia E., Battaglia F. (2017). Academic stress disrupts cortical plasticity in graduate students. Stress.

[6] Putwain D.W. (2011). How is examination stress experienced by secondary students preparing for their General Certificate of Secondary Education examinations and how can it be explained?. Int. J. Qual. Stud. Educ..

[7] Meijer J. (2007). Correlates of student stress in secondary education. Educ. Res..

[8] Clemente-Suárez V.J., Beltrán-Velasco A.I., Bellido-Esteban A., Ruisoto-Palomera P. (2018). Autonomic Adaption to Clinical Simulation in Psychology Students: Teaching Applications. Appl. Psychophysiol. Biofeedback.

[9] Beltrán-Velasco A.I., Ruisoto-Palomera P., Bellido-Esteban A., García-Mateos M., Clemente-Suárez V.J. (2019). Analysis of Psychophysiological Stress Response in Higher Education Students Undergoing Clinical Practice Evaluation. J. Med. Syst..

[10] Beltrán-Velasco A.I., Bellido-Esteban A., Ruisoto-Palomera P., Clemente-Suárez V.J. (2018). Use of Portable Digital Devices to Analyze Autonomic Stress Response in Psychology Objective Structured Clinical Examination. J. Med. Syst..

[11] Gómez B., Escobar A. (2002). Neuroanatomía del estrés. Rev. Mex. Neurocienc..

[12] Valizadeh L., Farnam A., Rahkar Farshi M. (2012). Investigation of Stress Symptoms among Primary School Children. J. Caring Sci..

[13] Bücker S., Nuraydin S., Simonsmeier B.A., Schneider M., Luhmann M. (2018). Subjective well-being and academic achievement: A meta-analysis. J. Res. Pers..

[14] Wang C., Li H., Lockard B. (2018). Comparison of Three Modes of Exercise in Reducing Stress and Improving Self-Esteem among Underserved Children at Title I Elementary Schools. Heal. Sci. J..

[15] Faught E.L., Ekwaru J.P., Gleddie D., Storey K.E., Asbridge M., Veugelers P.J. (2017). The combined impact of diet, physical activity, sleep and screen time on academic achievement: A prospective study of elementary school students in Nova Scotia, Canada. Int. J. Behav. Nutr. Phys. Act..

[16] Kim Y., Kwak K., Lee S. (2016). Does Optimism Moderate Parental Achievement Pressure and Academic Stress in Korean Children?. Curr. Psychol..

[17] Jovarini N., Barbosa V., Correia-Zanini M. (2018). Influencia de las habilidades sociales y factores de estrés sobre lo rendimiento escolar el 6^o^ año. Paidéia.

[18] Oberle E., Schonert-Reichl K.A. (2016). Stress contagion in the classroom? The link between classroom teacher burnout and morning cortisol in elementary school students. Soc. Sci. Med..

[19] James A. (2011). Managing Stress in Education: A Comprehensive Guide for Staff and Students.

[20] Suresh Prabu P. (2015). A Study on Academic Stress among Higher Secondary Students. Int. J. Humanit. Soc. Sci. Invent..

[21] Zhang X., Yan H., Yu H., Zhao X., Shah S., Dong Z., Yang G., Zhang X., Muse T., Li J. (2019). Childhood Urbanization Affects Prefrontal Cortical Responses to Trait Anxiety and Interacts With Polygenic Risk for Depression. Biol. Psychiatry.

[22] Hodson C.B., Sander H.A. (2017). Green urban landscapes and school-level academic performance. Landsc. Urban Plan..

[23] Kweon B.-S., Ellis C.D., Lee J., Jacobs K. (2017). The link between school environments and student academic performance. Urban For. Urban Green..

[24] Valadez A.A., Bravo M.C., Vaquero J.E. (2019). Urban stressors, stress and coping strategies in inhabitants of Mexico city. Rev. Electr. Psicol. Iztacala.

[25] Hoffmann B., Nieuwenhuijsen M., Khreis H. (2018). Air pollution in cities: Urban and transport planning determinants and health in cities. Integrating Human Health into Urban and Transport Planning: A Framework.

[26] Mouratidou K., Karamavrou S., Karatza S., Schillinger M. (2020). Aggressive and socially insecure behaviors in kindergarten and elementary school students: A comparative study concerning gender, age and geographical background of children in Northern Greece. Soc. Psychol. Educ..

[27] Laurel Whalen E.E.C. (2015). Understanding Stress and Aggression Behaviors among Urban Youth. J. Yoga Phys. Ther..

[28] Sher J.P. (2019). Rural Education In Urbanized Nations: Issues And Innovations.

[29] Harris F. (2017). The nature of learning at forest school: Practitioners’ perspectives. Education.

[30] Clemente-Suárez V.J., Fernandes R.J., Arroyo-Toledo J.J., Figueiredo P., González-Ravé J.M., Vilas-Boas J.P. (2015). Autonomic adaptation after traditional and reverse swimming training periodizations. Acta Physiol. Hung..

[31] Malik M., Bigger J.T., Camm A.J., Kleiger R.E., Malliani A., Moss A.J., Schwartz P.J. (1996). Heart rate variability: Standards of measurement, physiological interpretation, and clinical use. Eur. Heart J..

[32] Tornero-Aguilera J.F., Robles-Pérez J.J., Clemente-Suárez V.J. (2018). Use of psychophysiological portable devices to analyse stress response in different experienced soldiers. J. Med. Syst..

[33] Delgado R., Robles-Pérez J., Aznar S., Clemente-Suárez V. (2018). Inalambric Biofeedback Devices to Analyze Strength Manifestation in Military Population. J. Med. Syst..

[34] Janz K., Lutuchy E., Wenthe P., Levy S. (2008). Measuring Activity in Children and Adolescents Using Self-Report: PAQ-C and PAQ-A. Med. Sci. Sports Exerc..

[35] Castrillón Moreno D.A., Borrero Copete P.E. (2005). Validación Del Inventario De Ansiedad Estado—Rasgo (Staic) En Niños Escolarizados Entre Los 8 Y 15 Años. Acta Colomb. Psicol..

[36] Kim H.G., Cheon E.J., Bai D.S., Lee Y.H., Koo B.H. (2018). Stress and heart rate variability: A meta-analysis and review of the literature. Psychiatry Investig..

[37] Corraliza J., Collado S. (2015). La naturaleza cercana como moderadora del estrés infantil. Psicothema.

[38] Lee M.-S., Eum K.-D., Fang S., Rodrigues E., Modest G., Christiani D. (2014). Oxidative Stress and Systemic Inflammation as Modifiers of Cardiac Autonomic Responses to Particulate Air Pollution. Int. J. Cardiol..

[39] Krämer B., Diekhof E., Gruber O. (2017). Effects of city living on the mesolimbic reward system-An fmri study: Effects of City Living on the Mesolimbic Reward System. Hum. Brain Mapp..

[40] Burns R.A., Machin M.A. (2013). Employee and Workplace Well-being: A Multi-level Analysis of Teacher Personality and Organizational Climate in Norwegian Teachers from Rural, Urban and City Schools. Scand. J. Educ. Res..

[41] Abdel Rasoul G., Al-Batanony M., Mahrous O., Abo-Salem M., Gabr H. (2012). Environmental Lead Exposure among Primary School Children in Shebin El-Kom District, Menoufiya Governorate, Egypt. Int. J. Occup. Environ. Med..

[42] Calderón-Garcidueñas L., Medina-Cortina J., Mora-Tiscareño A. (2012). Impacto de la contaminación ambiental en el niño clínicamente sano. Acta Pediátrica México.

[43] Hatch G.E. (2010). Pollution and oxidative stress in schoolchildren. Indian Pediatr..

[44] Alvarsson J.J., Wiens S., Nilsson M.E. (2010). Stress recovery during exposure to nature sound and environmental noise. Int. J. Environ. Res. Public Health.

[45] Clemente-Suárez V.J. (2020). Multidisciplinary intervention in the treatment of mixed anxiety and depression disorder. Physiol. Behav..

[46] CINFA (2017). VII Estudio CINFASALUD: “Percepción y Hábitos de la Población Española en Torno al Estrés".

[47] Tishukaj F., Shalaj I., Gjaka M., Ademi B., Ahmetxhekaj R., Bachl N., Tschan H., Wessner B. (2017). Physical fitness and anthropometric characteristics among adolescents living in urban or rural areas of Kosovo. BMC Public Health.

[48] Walhain F., Van Gorp M., Lamur K.S., Veeger D.H.E.J., Ledebt A. (2016). Health-related fitness, motor coordination, and physical and sedentary activities of urban and rural children in Suriname. J. Phys. Act. Heal..

[49] Wojtyła-Buciora P., Stawińska-Witoszyńska B., Wojtyła K., Klimberg A., Wojtyła C., Wojtyła A., Samolczyk-Wanyura D., Marcinkowski J.T. (2014). Assessing physical activity and sedentary lifestyle behaviours for children and adolescents living in a district of Poland. what are the key determinants for improving health?. Ann. Agric. Environ. Med..

[50] Niemistö D., Finni T., Haapala E.A., Cantell M., Korhonen E., Sääkslahti A. (2019). Environmental correlates of motor competence in children-the skilled kids study. Int. J. Environ. Res. Public Health.

[51] Dario N., Hrvoje P., Branislav A. (2010). Are rural children really in better shape? Case of Croatia. Fit. Perf. J..

[52] Bruner M.W., Lawson J., Pickett W., Boyce W., Janssen I. (2008). Rural Canadian adolescents are more likely to be obese compared with urban adolescents. Int. J. Pediatr. Obes..

[53] Chwałczyńska A., Rutkowski T., Jȩdrzejewski G., Wójtowicz D., Sobiech K.A. (2018). The Comparison of the Body Composition of Children at the Early School Age from Urban and Rural Area in Southwestern Poland. Biomed Res. Int..

[54] Strochlic R., Au L., Ritchie L. (2017). Is urban-rural location associated with weight status in school children? An examination of 42 small and rural Californian counties. Rural Remote Health.

[55] McIsaac J.L.D., Kirk S.F.L., Kuhle S. (2015). The association between health behaviours and academic performance in Canadian elementary school students: A cross-sectional study. Int. J. Environ. Res. Public Health.

[56] Tognarelli M., Picciolli P., Vezzosi S., Isola A., Moretti F., Tommassetto E., Fantuzzi A.L., Bedogni G. (2004). Nutritional status of 8-year-old rural and urban Italian children: A study in Pistoia, Tuscany. Int. J. Food Sci. Nutr..

[57] Cutumisu N., Traoré I., Paquette M.C., Cazale L., Camirand H., Lalonde B., Robitaille E. (2017). Association between junk food consumption and fast-food outlet access near school among Quebec secondary-school children: Findings from the Quebec Health Survey of High School Students (QHSHSS) 2010-11. Public Health Nutr..

[58] Schetzina K.E., Dalton W.T., Lowe E.F., Azzazy N., Vonwerssowetz K.M., Givens C., Stern H.P. (2009). Developing a coordinated school health approach to child obesity prevention in rural Appalachia: Results of focus groups with teachers, parents, and students. Rural Remote Health.

[59] Badia Martín M., Clariana Muntada M., Gotzens Busquets C., Cladellas Pros R., Dezcallar Sáez T. (2015). Video games, television and academic performance in elementary school estudents. Pixel-Bit. Rev. Medios Educ..

[60] Wingfield R.J., McNamara J.P.H., Janicke D.M., Graziano P.A. (2011). Is there a relationship between body mass index, fitness, and academic performance? mixed results from students in a southeastern united states elementary school. Curr. Issues Educ..

[61] He J., Chen X., Fan X., Cai Z., Huang F. (2019). Is there a relationship between body mass index and academic achievement? A meta-analysis. Public Health.

[62] González M.J., Díaz-Giráldez F., Martín I., Delgado M., Trianes M.V. (2016). Estrés Cotidiano Y Precisión Lectora En Niños De Educación Primaria. Int. J. Dev. Educ. Psychol. Rev. INFAD Psicol..

[63] Hansstein F.V., Hong Y., Di C. (2017). The relationship between new media exposure and fast food consumption among Chinese children and adolescents in school: A rural–urban comparison. Glob. Health Promot..

[64] Daly C.M., Foote S.J., Wadsworth D.D. (2017). Physical Activity, Sedentary Behavior, Fruit and Vegetable Consumption and Access: What Influences Obesity in Rural Children?. J. Community Health.

[65] Yulianti K., Denessen E. (2018). The effects of parental invol vement on childrens’ education: A study in elementary schools in Indonesia. Int. J. Parents Educ..

[66] Koszycki D., Taljaard M., Bielajew C., Gow R.M., Bradwejn J. (2019). Stress reactivity in healthy child offspring of parents with anxiety disorders. Psychiatry Res..

[67] Sheffield A., Cui L., Criss M., Simmons W.K., Cole P., Hollenstein T. (2018). Emotion Regulation Dynamics During Parent–Child Interactions. Emotion Regulation. A Matter of Time.

[68] Shaffer F., Ginsberg J.P. (2017). An Overview of Heart Rate Variability Metrics and Norms. Front. Public Heal..

[69] Rekawek J., Miszczak-Knecht M., Kawalec W., Mielniczuk J. (2003). Heart rate variability in healthy children. Folia Cardiol..

[70] de Godoy M.F., Lima M., Aslanidis T. (2019). Evolution of Parasympathetic Modulation throughout the Life Cycle. Autonomic Nervous System Monitoring.

[71] Guenther P., Juan W., Lino M., Hiza H., Fungwe T., Lucas R. (2008). Diet Quality of Low-Income and Higher Income Americans in 2003-04 as Measured by the Healthy Eating Index-2005. FASEB J..

[72] Stuber N. (2014). Nutrition and Students’ Academic Performance. How does nutrition influence students’ academic. Wilder Res..

[73] World Health Organization (2016). Food and Nutrition Policy for Schools: A Tool for the Development of School Nutrition Programmes in the European Region.

